# Serologic evolution and follow-up to IgG antibodies of infants born to mothers with gestational COVID

**DOI:** 10.1186/s12884-023-05926-6

**Published:** 2023-08-30

**Authors:** Sara Vigil-Vázquez, Ángela Manzanares, Alicia Hernanz-Lobo, Itziar Carrasco-García, Clara Zamora del Pozo, Alba Pérez-Pérez, Elena María Rincón-López, Begoña Santiago-García, María del Pilar Pintado-Recarte, Roberto Alonso-Fernández, Manuel Sánchez-Luna, María Luisa Navarro-Gómez

**Affiliations:** 1 Department of Neonatology, Hospital General Universitario Gregorio Marañón, Calle O’Donnel 48, Madrid, 28009 Spain; 2 Pediatric Infectious Disease Unit, Instituto de Investigación Sanitaria Gregorio Marañón, Madrid, Spain; 3 Department of Pediatrics Infectious Diseases, Hospital General Universitario Gregorio Marañón, Madrid, Spain; 4 CIBERINFEC, Instituto de Salud Carlos III, Madrid, Spain; 5 Department of Obstetrics and Gynecology, Hospital General Universitario Gregorio Marañón, Madrid, Spain; 6 Department of Clinical Microbiology and Infectious Diseases, Hospital General Universitario Gregorio Marañón, Madrid, Spain; 7 Facultad de Medicina, Universidad Complutense de Madrid, Madrid, Spain

**Keywords:** Gestational COVID, Neonatal SARS-COV-2 infection, Serological test, Antibodies titers, Antibodies transplacental transfer

## Abstract

**Background:**

It is known that SARS-CoV-2 antibodies from pregnant women with SARS-CoV-2 infection during pregnancy cross the placenta but the duration and the protective effect of these antibodies in infants is scarce.

**Methods:**

This prospective study included mothers with SARS-COV-2 infection during pregnancy and their infants from April 2020 to March 2021. IgG antibodies to SARS-CoV-2 spike protein were performed on women and infants at birth and at two and six months during follow-up. Anthropometrical measures and physical and neurological examinations and a clinical history of symptoms and COVID-19 diagnosis were collected. Simple linear regression was performed to compare categorical and continuous variables. To compare the mother’s and infant’s antibody titers evolution, a mixed linear regression model was used. A predictive model of newborn antibody titers at birth has been established by means of simple stepwise linear regression.

**Results:**

51 mother-infant couples were included. 45 (90%) of the mothers and 44 (86.3%) of the newborns had a positive serology al birth. These antibodies were progressively decreasing and were positive in 34 (66.7%) and 7 (13.7%) of infants at 2 and 6 months, respectively. IgG titers of newborns at birth were related to mothers’ titers, with a positive moderate correlation (Pearson’s correlation coefficient: 0.82, p < 0,001). Fetal/maternal antibodies placental transference rate was 1.3 (IQR: 0.7–2.2). The maternal IgG titers at delivery and the type of maternal infection (acute, recent, or past infection) was significantly related with infants’ antibody titers at birth. No other epidemiological or clinical factors were related to antibodies titers. Neurodevelopment, psychomotor development, and growth were normal in 94.2% of infants in the third follow-up visit. No infants had a COVID-19 diagnosis during the follow-up period.

**Conclusions:**

Transplacental transfer of maternal antibodies is high in newborns from mothers with recent or past infection at delivery, but these antibodies decrease after the first months of life. Infant’s IgG titers were related to maternal IgG titers at delivery. Further studies are needed to learn about the protective role of maternal antibodies in infants.

## Background

The SARS-CoV-2 pandemic has rapidly spread worldwide in the last two years, and today the numbers are as high as more than 200 million confirmed cases and 5 million deaths [1]. During this time, one of the main concerns to health care workers has been special populations such as pregnant women and their infants, due to their high susceptibility to severe infections and the possible outcomes that COVID-19 infection during pregnancy could have in infants.

It is widely known that transplacental acquired antibodies are a key element for the protection of the newborn against infections [2, 3]. It has been demonstrated that SARS-CoV-2 antibodies from pregnant women with SARS-CoV-2 infection during pregnancy cross the placenta, and have been detected in cord blood [4–7]. Nevertheless, it has been suggested that this transfer is lower if the infection occurs close to birth time, and less efficient than expected if compared with other pathogens [8–10]. The duration of these antibodies in infants is a scarcely studied issue with important consequences for newborns, as COVID-19 infection is more severe in neonates than in older children, and especially taking into account that it is unlikely that SARS-CoV-2 vaccines would be approved for young infants in the near future [11]. Small samples indicate that maternal antibodies’ titers rapidly decrease in the first weeks of life, although they may persist in a low proportion of infants at 6 months of life [12–15]. The protective effect that SARS-CoV-2 maternal antibodies could have on infant’s infection has not yet been described.

Data on perinatal outcomes of infants born to mothers with SARS-CoV-2 infection during pregnancy is rising [16]. In the first wave on COVID pandemic, poor neonatal outcomes have been shown, with high rates of cesarean sections, preterm infants, respiratory distress and NICU admission, especially in high-risk population [2, 17–24]. Over time, improved treatment of pregnant women with COVID resulted in decreased cesarean section rates and overall adverse perinatal outcomes [25].

Vertical transmission of SARS-CoV-2 has been demonstrated, usually with low rates and mild or no symptoms [21, 26–30]. Horizontal transmission in the first weeks of life seems to be also low in these infants, with scant clinical manifestations [9, 31].

The main objective of our study was to describe the maternal antibody response to SARS CoV-2 during pregnancy and the transplacental passage of the passive IgG to their newborns and the duration of these antibodies in these infants until 6 months. The secondary aim was to describe if maternal antibodies have a protective effect against neonatal infection.

## Methods

We conducted a prospective study at Gregorio Marañón University Hospital, a tertiary hospital in Madrid (Spain). We included mother-infants’ dyads from the Spanish multicenter GESNEO-COVID cohort study. We included the births from April 2020 to March 2021. During the inclusion period, no mother had been vaccinated against SARS-COV-2.

GESNEO-COVID cohort recruited mothers with SARS-COV-2 infection during pregnancy and their infants. Anti SARS-COV-2 IgG antibodies were performed on women and infants at three different moments: at birth (cord or neonatal blood or plasma samples for infants), and at two and six months of the infant’s life. Only couples with all serological samples collected were included in this study.

COVID-19 infection was diagnosed in women if they had either a positive result in a reverse transcription polymerase chain reaction (RT-PCR) done on the nasopharyngeal swab (NPS) during pregnancy or a positive serology at delivery. A screening RT-PCR and SARS-CoV2 IgG antibodies were done for all women admitted to the hospital at delivery.

Data on maternal infection were collected retrospectively from electronic medical records. The date of infection was considered the day of COVID-19 related symptoms onset, or the first positive RT-PCR results in asymptomatic women. Infections in women with a serological diagnosis and who were asymptomatic could not be dated.

Pregnant women were classified according to SARS-COV-2 microbiological test at delivery into 3 groups: acute infection (positive RT-PCR, negative IgG antibodies), recent infection (positive RT-PCR, positive IgG antibodies), and past infection (negative RT-PCR at delivery, with positive RT-PCR during pregnancy and/or positive IgG antibodies at delivery).

Following the recommendations of the Spanish Society of Neonatology and the World Health Organization and according to our center’s protocol, asymptomatic newborns whose mothers had an appropriate clinical condition were rooming-in in obstetrics wards and breastfeeding was promoted [32–34]. A RT-PCR on a NPS was performed on newborns in their first 48 h of life and repeated in the next 24 h only if positive.

Three follow-up visits were performed to infants. The first one at 15 days of life, the second one at two months of life and the third one at six months of life. Anthropometrical measures and physical and neurological examinations were performed by a pediatrician. COVID-19 related symptoms, hospital admissions and RT-PCR tests were recorded. A RT-PCR was performed at the first visit. Serological tests were done in the second and third visits. Serological tests were also performed on mothers in the same study visits. Vaccination of the mothers during the follow up was recorded.

Serological tests in serum samples were performed to detect IgG antibodies against the spike protein of SARS-COV-2. These tests were carried out by using a quantitative chemiluminescent assay (SARS-CoV-2 IgG II Quant Reagent Kit) and an ARCHITECT i2000 instrument (Abbott; Chicago, USA). IgG levels were expressed in AU/mL (arbitrary units per millilitre) and were converted to BAU/mL (binding antibody units per millilitre) using the conversion coefficient provided by the manufacturer (1 BAU = 0.142 X AU) to standardize the results. The linear detection range was from 0 to 5,680 BAU/mL, being results above 7.10 BAU/mL considered positive.

RT-PCR tests on NPS samples were carried out to detect the N gene and the *ORF1a1b* gene (TaqPath Multiplex, Thermo Fisher®).

### Statistical analysis

Continuous variables were presented with median and interquartile ranges (IQR). Categorical variables were presented as total counts and percentages (%). Due to the wide range of values, antibody titers have been expressed by logarithmic scale transformation in base 10 to normalize them.

Transplacental transfer ratio was calculated as infant IgG concentration divided by maternal IgG concentration at birth. Correlations between maternal and neonatal IgG concentrations and between transplacental transfer ratio were reported using the Pearson correlation coefficient (r).

Simple linear regression was performed to compare categorical and continuous variables. A p-value < 0.05 was considered statistically significant. To compare the mother’s and infant’s antibody titers evolution, a mixed linear regression model was used.

A predictive model of newborn antibody titers at birth has been established. The selection of the variables to be introduced in the model was made following clinical and statistical criteria by means of simple stepwise linear regression minimizing Akaike Information Criterion (AIC), Bayes Information Criterion (BIC) and R2-adjusted.

Data were analyzed using StataCorp. 2019. *Stata Statistical Software: Release 16*. College Station, TX: StataCorp LLC and R vs. 4.1.3.

## Results

Of 266 women attended with SARS-COV-2 infection during pregnancy or delivery during the study period, 51 mother-infant dyads were included. Demographic and clinical characteristics of mothers and infants are described in Tables 1 and 2 respectively.

**Table 1:** Demographic and clinical characteristics of pregnant women

	Pregnant women n = 51
Age (years)	33.8 (29.5–36.4)
Ethnicity	
- Caucasian - Latino American - Arabic	24 (47.1%) 25 (49.0%) 2 (3.9%)
Diagnostic test	
- RT-PCR in NF swab - Serological test	42 (82.4) 9 (17.6%)
GA at diagnosis (w)	35.2 (28.2–38.4)
Any comorbidity	8 (15.7%)
Symptomatology	26 (51.0%)
Respiratory symptoms severity*	
- Mild - Moderate - Severe	9 (64.3%) 5 (35.7%) 0 (0%)
Pneumonia COVID-19	5 (9.8%)
Specific treatment	4 (7.8%)
COVID hospitalization	5 (9.8%)
- ICU admission	0 (0%)
Days of admission	6 (6–6)
RT-PCR + at delivery	17 (33.3%)
Infection classification at delivery	
- Acute infection - Recent infection - Past infection	5 (9.8%) 12 (23.5%) 34 (66.7%)

Severity of respiratory infection was stratified into mild (upper respiratory tract symptoms), moderate (pneumonia confirmed by chest X-ray without signs of severity) and severe (presence of hypoxemia with partial oxygen saturation [SatO2] < 90%, acute confusional state, or arterial hypotension)

Continuous variables are described as the median and interquartile range (IQR). Categorical variables as absolute frequencies and percentages

GA: gestational age, ICU: intensive care unit

**Table 2:** Demographic and clinical characteristics of infants

	Infants n = 51
Sex (male)	28 (54.9%)
GA (w)	39.6 (38.4–40.3)
Prematurity rate	2 (3.9%)
Somatometric characteristics	
- Weight (g) - Length (cm) - Head circumference (cm)	3300 (3010–3520) 50 (49–51) 34 (33.5–35)
Type of delivery	
- Eutocic - Instrumental - Cesarean section	39 (76.5%) 7 (13.7%) 5 (9.8%)
Apgar	
- 1’ - 5’	9 (9–9) 10 (10–10)
Symptomatology	5 (9.8%)
- Jaundice - Perinatal asphyxia and HIE - TTNB	4 (7.8%) 1 (2.0%) 1 (2.0%)
Need for NICU admission	1 (2.0%)
Breastfeeding method	
- Maternal - Artificial - Mixed	32 (62.7%) 3 (5.9%) 16 (31.4%)

Continuous variables are described as medians and interquartile ranges (IQR). Categorical variables are presented as absolute frequencies and percentages

GA: gestational age, NICU: neonatal intensive care unit, HIE: hypoxic-ischemic encephalopathy, TTNB: transient tachypnea of the newborn

SARS-CoV-2 RT-PCR was performed on 72.6% (37) of newborns in the first 48 h of life, and they were all negative. All infants completed the follow-up visits. No infants had a COVID-19 diagnosis during the follow-up period. 4.3% (2) of the infants had a cohabitant with a COVID-19 diagnosis. One (1.96%) infant needed hospital admission due to a brief resolved unexplained event (BRUE). Neurodevelopment, psychomotor development, and growth were normal in 94.2% of infants in the third follow-up visit. One infant with Down syndrome had psychomotor development retardation at 6 months follow-up, one infant had growth delay and another had mild microcephaly with normal imaging tests.

**Fig. 1:**
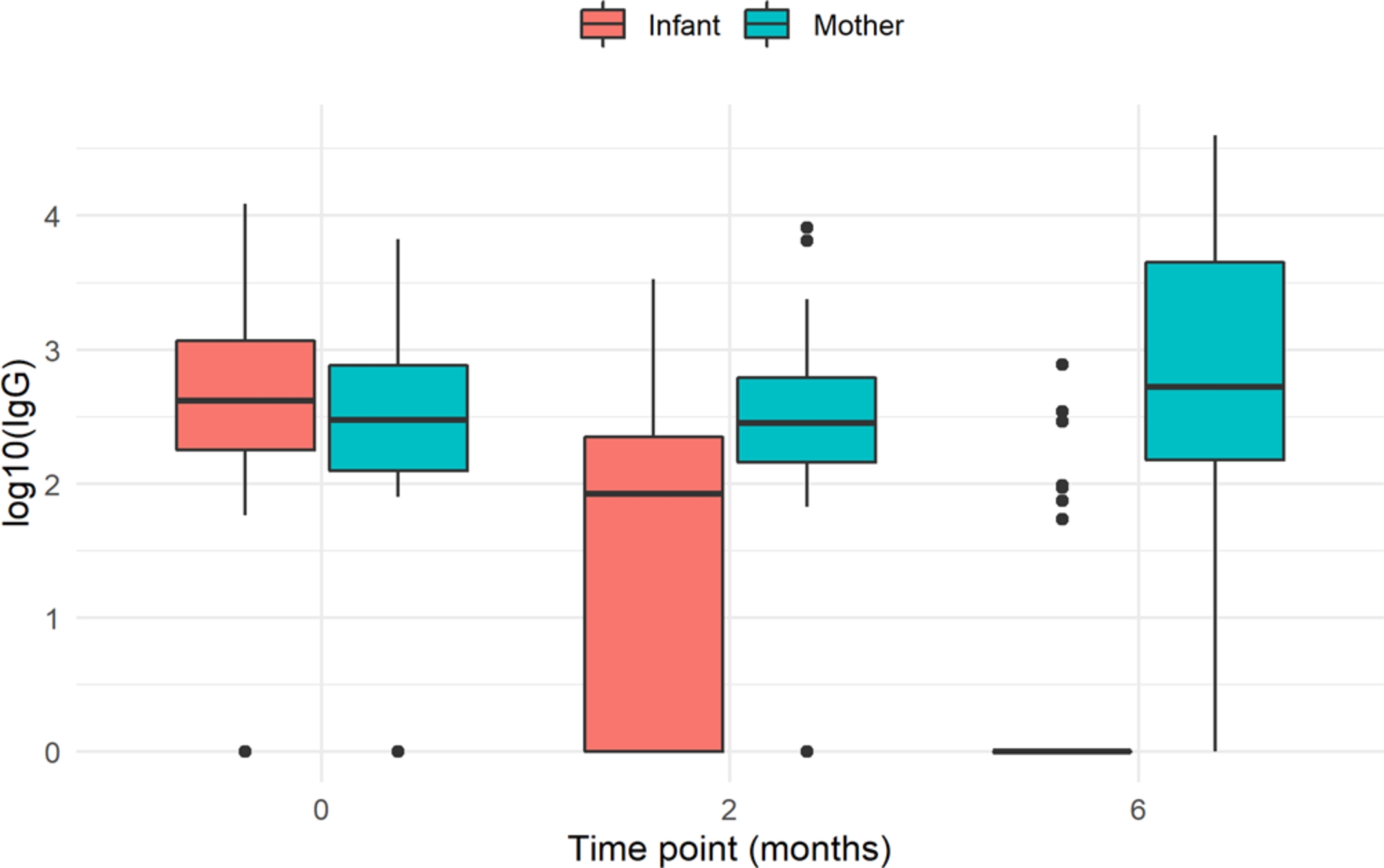
Box-and-whisker plot for log10 IgG values between mothers and newborns

The first blood analysis was done at a median of 2.1 (IQR 2.0-2.6) months and the second one at a median of 6.1 (IQR: 6-6.5) months. Almost 90% (45) of the mothers had positive IgG antibodies at delivery. At birth, 86.3% (44) of the newborns had positive IgG antibodies in cord or neonatal blood. These results were progressively decreasing, and antibodies were positive in 66.7% (34) and 13.7% (7) of infants at 2 and 6 months, respectively. The evolution of IgG antibody titers in mothers and infants during follow-up is represented in Fig. 1.

29% (15) of the women received SARS-CoV-2 vaccination between the second and the third follow-up visits. Despite the difference in maternal titers, no difference in titers at 6 months was found in newborns whose mothers had been vaccinated (39.9 AU/mL vs. 19.4 AU/mL, p = 0.596).

**Fig. 2:**
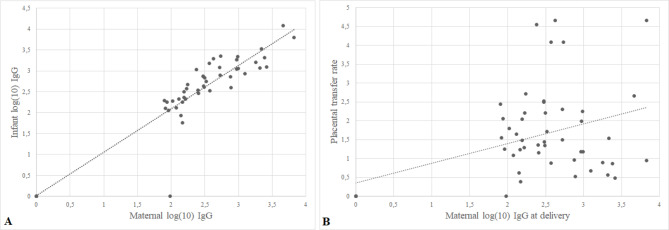
Scatter plots and correlation lines. (A) Infants IgG antibodies levels at birth according to maternal IgG antibodies levels. (B) Placental transfer ratio according to maternal IgG antibody levels

IgG titers of newborns at birth were related to mothers’ titers at delivery, with a positive moderate correlation (Pearson’s correlation coefficient: 0.82, CI95% 0.70–0.98, p < 0,001). Fetal/maternal antibodies placental transference rate was 1.3 (IQR: 0.7–2.2). Scatter plots and correlation lines are shown in Fig. 2.

At 2 months study visit, infants experienced a 1 unit decrease in log10 IgG antibodies titers compared to their mothers (IC95% -1.4− -0.63, p < 0.001), and at 6 months, a 2.6 decrease (IC95% -2.9― -2.2, p < 0.001).

At delivery, 9.8% (5) of the women had an acute SARS-CoV-2 infection, 23.5% (12) had a recent infection, and 66.7% (34) had a past infection. All the infants born to mothers with acute infection at delivery had negative serologies at birth and during the follow up. There were another two newborns with a negative serology at birth. One of them was a newborn from a mother with recent infection and low antibodies titers at delivery (96 UA/mL), and the other one was a newborn from a mother with an asymptomatic infection at 34 weeks of pregnancy who had not developed antibodies.

The maternal IgG antibodies titers at delivery and type of maternal infection was significantly related in a univariant analysis with infants’ antibody titers at birth. No other epidemiological or clinical factors were related to antibodies titers. These results are shown in Table 3.

**Table 3:** Univariate analysis by simple linear regression for log(10) IgG at birth

Variable	Beta	95% CI^1^	p-value
Maternal age (years)	0.11	-0.04, 0,06	0.688
Ethnicity			
Caucasian Latino American Arabic	— 0.17 0.13	— -1.44, 1,78 -0.49, 0.76	0.837 0.668
GA at diagnosis (weeks)	0.00	-0.00, 0.00	0.482
Any comorbidity	0.33	-0.50, 1.16	0.427
Symptomatology	0.03	-0.57, 0.64	0.911
Specific treatment	0.81	-0.29, 1.92	0.147
Hospital admission	0.84	-0.16, 1.83	0.097
Infection classification at delivery			
Acute infection Recent infection Past infection	— 2.51 2.70	— 1.73, 3.28 2.00, 3.40	**< 0.001** **< 0.001**
Maternal IgG titers at delivery (AU/mL)	2.71	2.18, 3.24	**< 0.001**
GA at delivery (weeks)	0.06	-0.19, 0.30	0.645
Sex (male)	-0.31	-0.91, 0.30	0.312
Type of delivery			
Eutocic Instrumental Cesarean section	— -0.90 -0.22	— -0.99, 0.81 -1.26, 0.82	0.840 0.671
Breastfeeding method			
Maternal Artificial Mixed	— -0.74 0.32	— -2.03, 0.55 -0.33, 0.96	0.255 0.328

^1^CI = Confidence Interval

The best-established model for predicting antibody titers at birth has included the maternal IgG antibodies titers at delivery and the need for hospitalization. The IgG antibodies titers at the birth of the newborns were statistically significantly related in a multiple linear regression to the maternal IgG antibodies titers at delivery. Although maternal hospitalization is not statistically significant, it has been kept in the model because including it improves its fit. The adjusted model with its coefficients and 95%CI is shown in Table 4.

**Table 4:** Multivariate linear regression for log10(IgG) of the newborn as a function of the variables selected by statistical criteria at birth

Variable	Beta	95% CI^1^	p-value
Maternal IgG titers at delivery (AU/mL)	2.7	2.13, 3.18	**< 0.001**
Hospital admission	0.5	-0.1, 1.1	0.09

^1^CI = Confidence Interval

AIC: 72.7, BIC: 77.9, R2 adj: 0,685

## Discussion

In our sample, 86.3% of the newborns born to mothers with SARS-CoV-2 infection during pregnancy had positive anti SARS-COV-2 IgG antibodies at birth, and these antibodies progressively declined during the first 6 months of life. The timing of maternal infection was the only factor associated with antibody titers in the infants.

Previous publications analyzing the transplacental transfer of SARS-CoV-2 maternal antibodies described rates of newborns with positive antibodies at birth are similar to ours, varying from 62 to 91% [4, 5, 10]. These variations could be explained by the serological status of the mothers at delivery, which was different in the different studies depending on the inclusion criteria. In our sample, the percentage of infants with positive serologies at birth was as high as 95.6% if the mother had a positive serology at delivery. As previously described, infant’s IgG titers were related to maternal IgG titers [14, 35, 36].

In our sample, the only other factor associated with antibody titers in the newborn was the timing of maternal infection. These data are in concordance with data published by Flannery et al. In their study they analyze 83 pregnant women with positive SARS-CoV-2 serologies (IgG or IgM) and found all cord blood samples had positive IgG if the mother had the infection for more than 17 days prior to delivery [4]. On the contrary, no difference in newborns antibody titers was found if the infection was more or less than 14 days prior to delivery in the study conducted by Joseph et al. [5]. It is to notice that all women in their sample had a positive IgG at delivery, so they wouldn’t be classified as acute infection in our cohort.

We found that newborns from mothers with acute infection at delivery had negative antibodies, as described by Conti. et al. [9]. Mothers with acute infection at delivery had not yet developed an adaptative immune response with IgG antibody production, so these antibodies could not be transferred to their newborns; as these infants did not have a SARS-CoV-2 infection, they did not develop their own antibodies during the follow up. The situation of the newborn with maternal infection in the 34th week of pregnancy who did not present antibodies could be considered as a special case in which the mother did not develop antibodies, or it could also represent a false positive in the diagnostic test of the pregnant woman.

Other factors have been associated with antibody titers, such as maternal symptoms or hospitalization [5]. Nevertheless, and as seen in other cohorts, we have not found this relation [4, 10].

The duration of maternal antibodies in infants has been scarcely studied. Gao et al. described a rapid decline in the first 75 days of life of SARS-CoV-2 IgG titers in 11 infants with positive SARS-CoV-2 serologies at birth [13]. Song et al. also described a decrease in SARS-CoV-2 IgG titers in the first 28 weeks of life in 48 infants with positive SARS-CoV-2 serologies at birth [12]. Also, Capretti et al. found that SARS-CoV-2 IgG level progressively decreased in all infants in their sample, with 97% infants seronegative at 6 months of age [37]. In our sample, of 43 infants with SARS-CoV-2 IgG at delivery, 9 (20.9%) had lost these antibodies at 2 months of life and only 7 (16.2%) maintained them at 6 months of life. This makes it necessary to investigate vaccination strategies for infants above 6 months, when maternal protection is lost, and the risk of infection increases.

In contrast with other studies, in our cohort transplacental transfer of maternal antibodies was appropriate, with a 1.33 ratio [5, 10]. This is significantly higher than previously described for SARS-CoV-2, and similar to the ratio described for other infections such as *Bordetella pertussis*, influenza or measles, so it could confer adequate protection against SARS-CoV-2 [2, 38, 39].

In our cohort, only 72% of infants were screened with PCR at birth. According to our center’s protocol, all newborns whose mothers have had an acute or recent infection at the time of delivery were screened with PCR at birth. When the mother had had a past infection at some time during pregnancy and was not infected at the time of delivery, it was not necessary to test with PCR on the newborn, and many parents refused to have it performed.

The rate of vertical transmission of SARS-CoV-2 has been reported to be very low in previous studies, as in our cohort, where no vertical-transmitted infections were found [21, 26, 27, 30]. The rates of horizontal transmission have also been reported to be low, and we neither found horizontal SARS-CoV-2 infections during the follow-up, although 5 mothers had an acute infection at delivery and 2 cohabitants also had COVID-19 infection during the follow-up [21, 40, 41]. In addition, these 7 infants were breast-feeding, which could confer additional protection. This supports our hypothesis that maternal antibodies could prevent infection in the newborn.

Cenamo et al. found that even in cases without vertical transmission of SARS-CoV-2 infection, the cord Interferon-γ was significantly lower in cord blood of SARS-CoV-2-positive mothers, suggesting that infection can affect the fetal microenvironment even without severe maternal symptoms [42]. In our study, only SARS-COV-2 IgG antibodies were analyzed and no immunity study was performed on mothers and newborns. It would have been interesting to know the cord levels of Interferon-γ in our infants since none of them subsequently developed infection.

Serologies performed in our study (IgG anti-Spike Protein Receptor Domain antibodies) are detected not only after COVID-19 but also after SARS-CoV-2 vaccination. In Spain, vaccination for pregnant women started in May 2021, so women included in our sample were not vaccinated during pregnancy. 29.4% of women were vaccinated after delivery, between 2 and 6 months-follow-up, so this affected maternal antibody titers in the last visit, which remain high. In our sample, maternal vaccination did not affect newborn antibody titers at the end of follow-up. Maternal antibody titers at delivery and infant’s titers were not biased by vaccination. Although there is some evidence that SARS-CoV2 vaccination during pregnancy provides antibodies in cord blood to infants, if the duration and protection of these antibodies are similar than the provided in natural infection has not been studied [43]. Another important issues to be clarified soon is if infections or vaccination before pregnancy provide protection to infants and if there are differences in these antibodies depending on the SARS-CoV-2 variant.

One of our strengths is that we have included only mother-infants’ dyads with all samples taken and complete follow-up to facilitate the interpretation of the results. The clinical follow-up of infants is another of the strengths of our work, showing that the evolution is normal and that from the point of view of clinical protection we did not find moderate-severe symptomatic COVID infections in the follow-up cohort. Although we have the disadvantage of a single-center study, which affects the external validity of the study, all samples were analyzed in the same laboratory with the same microbiological technique, providing homogeneity to the sample and allowing the evolution of antibody titers over time to be correctly assessed.

One of the weaknesses of our study is that in a few mothers the diagnosis of infection was made by serological test at delivery, so we do not know the exact time of infection during pregnancy. One of the objectives was to know the protective effect of maternal antibodies on SARS-COV-2 infection in infants. Regarding this aim, other limitations are the retrospective nature of the study, the absence of active surveillance of SARS-COV-2 exposure among infants, and the absence of a control group. Although we performed RT-PCR at 15 days of life to infants and asked about COVID-19 related symptoms in the follow-up visits, it is possible that asymptomatic infections could be misdiagnosed.

Another weakness of the study is that there was no measuring of neutralizing activity antibodies. It is well known that neutralizing antibodies are essential for preventing severe disease. A recent study has demonstrated a strong neutralizing and specific maternal IgG response following SARS CoV-2 infection; however, they observed a significant reduction in neutralizing activity between maternal blood and cord blood [5].

## Conclusions

In conclusion, transplacental transfer of maternal antibodies is high in newborns from mothers with recent or past infection at delivery, but these antibodies decrease quickly in the first months of life. Infant’s IgG titers were related to maternal IgG titers at delivery. Infants with antibodies may be protected against SARS-COV-2 infection.

## Data Availability

The datasets used and/or analyzed during the current study are available from the corresponding author on reasonable request.

## List of abbreviations

AIC: Akaike Information Criterion
BIC: Bayes information criterion
AU: Arbitrary units
BRUE: Brief resolved unexplained event
CI: Confidence interval
COVID-19: Coronavirus disease 2019
HGUGM: Gregorio Marañón University Hospital
IgG: Immunoglobulin G
IQR: Interquartile rate
NICU: Neonatal intensive care unit
NPS: Nasopharyngeal swab
RT-PCR: Reverse transcription polymerase chain reaction
SARS-CoV-2: Severe acute respiratory syndrome coronavirus
